# Sight and sound persistently out of synch: stable individual differences in audiovisual synchronisation revealed by implicit measures of lip-voice integration

**DOI:** 10.1038/srep46413

**Published:** 2017-04-21

**Authors:** Alberta Ipser, Vlera Agolli, Anisa Bajraktari, Fatimah Al-Alawi, Nurfitriani Djaafara, Elliot D. Freeman

**Affiliations:** 1 City, University of London, London, UK; 2 University of Sussex, Falmer, UK; 3 Middlesex University, London, UK; 4 Birkbeck, University of London, London, UK

## Abstract

Are sight and sound out of synch? Signs that they are have been dismissed for over two centuries as an artefact of attentional and response bias, to which traditional subjective methods are prone. To avoid such biases, we measured performance on objective tasks that depend implicitly on achieving good lip-synch. We measured the McGurk effect (in which incongruent lip-voice pairs evoke illusory phonemes), and also identification of degraded speech, while manipulating audiovisual asynchrony. Peak performance was found at an average auditory lag of ~100 ms, but this varied widely between individuals. Participants’ individual optimal asynchronies showed trait-like stability when the same task was re-tested one week later, but measures based on different tasks did not correlate. This discounts the possible influence of common biasing factors, suggesting instead that our different tasks probe different brain networks, each subject to their own intrinsic auditory and visual processing latencies. Our findings call for renewed interest in the biological causes and cognitive consequences of individual sensory asynchronies, leading potentially to fresh insights into the neural representation of sensory timing. A concrete implication is that speech comprehension might be enhanced, by first measuring each individual’s optimal asynchrony and then applying a compensatory auditory delay.

When people speak, we are used to seeing their lips moving at the same time as we hear them. Consequently, our understanding of speech can be greatly aided by the sight of concurrent lip movements^1^,^2^,^3^. Individuals with hearing impairment are particularly reliant on this^4^. Conversely poor lip-synch, as often found in streaming video and cable television, can impair speech comprehension and audiovisual integration^5^,^6^,^7^,^8^. Strangely however, our explicit perception of audiovisual synchrony does not always seem veridical^9^,^10^. This has been known since the late 18^th^ century, when astronomers timed the transit of celestial bodies relative to the sound of a ticking clock^11^,^12^,^13^. Consistent discrepancies between astronomers were noted, but could be compensated for by assigning each individual a unique ‘Personal Equation’ (PE) quantifying their perceptual asynchrony. These early observations led to the foundation of Experimental Psychology^14^, however researchers since have explained PEs as biases of attention or decision making^9^ or discarded them by averaging across individual differences. The present paper questions whether such a bias account provides a sufficient explanation, and explores the practical implications of perceptual asynchronies for interpretation of speech.

Much of the uncertainty surrounding interpretation of PEs stems from use of methods which estimate the physical asynchrony at which two events are perceived as synchronous (the point of subjective simultaneity; PSS), using explicit judgements of perceived temporal order, simultaneity or belongingness of two sensory events^10^,^15^,^16^,^17^. Though such measures may show retest stability reminiscent of PEs, discrepancies and low correlation between measurements of PSS obtained using different subjective methods have often been observed^18^,^19^,^20^,^21^. Such variability has been attributed to the influence of decisional variables^18^,^22^,^23^. In addition, subjective measures are readily swayed by attentional or decisional factors that may afford ‘prior entry’ of the selected sensory input to further processing or by biasing responses towards one modality or response^15^,^16^,^20^,^23^,^24^,^25^,^26^. Methods controlling for prior entry may still be susceptible to decisional biases^26^,^27^ and vice versa^28^. Less bias-prone measures of audiovisual timing do exist^29^,^30^,^31^,^32^, and have supported the notion of intrinsic sensory asynchrony^33^. However, the present study is the first to use implicit bias-resistant measures to examine normal individual differences in the asynchrony of audition relative to vision, and the reliability of such measures across retests. Our aims were to test whether implicit measures might show PE-like retest stability, and whether we have just one or multiple PEs for different tasks.

We used two implicit measures of perceptual asynchrony. One exploited the McGurk illusion^34^, in which incongruent lip movements and phonemes are combined to induce misheard phonemes; the other measured Degraded Speech identification, where accuracy typically benefits from congruent lip movements^1^. We measured these while varying audiovisual asynchrony^10^,^29^,^30^, and then for each individual estimated the asynchrony that maximised their McGurk effect and Degraded Speech identification accuracy (tMax). As assumed for explicit measures, the dependence of performance on audiovisual asynchrony might be informative about asynchronies intrinsic in the processing of auditory versus visual information and their integration; however note that it is not assumed that our implicit measures of audiovisual integration need relate directly to measures derived from explicit methods (such as the point of subjective synchrony), for different measures might probe different processes^18^,^19^,^20^,^21^,^35^. The advantage of the implicit approach is that it avoids response biases that readily affect explicit measures of subjective synchrony; the approach estimates sensory asynchrony indirectly by measuring the effects of physical (a)synchrony on objective measures of accuracy and audiovisual integration. Additionally, because they may probe preattentive integration mechanisms they are likely to be resistant to attentional biases or prior entry effects, at least under the low attentional load conditions employed here^36^,^37^,^38^.

Past investigations have found that measures of audiovisual synchronisation remain stable across repeated measurements, though they sometimes depended on measurement methods^10^,^11^,^12^,^13^. To assess whether our tMax values had similar trait-like stability over time, we compared performance across two repetitions of the same task, either within the same one-hour session, or one week apart. A comparison of tMax estimates across tasks also allows us to address the question of whether we each have just one or several Personal Equations each for different tasks. If tMax values correlated positively across tasks, this could be consistent with the role of common timing mechanisms^39^,^40^,^41^,^42^,^43^,^44^ underlying audiovisual integration; it might also be consistent with a gross effect of prior-entry speeding of one modality relative to another, or as a generic response bias^15^,^16^,^23^,^24^,^25^,^26^. Alternatively, a low correlation across tasks would be consistent with a network of distinct multimodal mechanisms for different tasks^45^,^46^,^47^,^48^,^49^, each subject to different processing and propagation latencies^10^,^17^,^27^,^50^.

Aside from the above theoretical implications, the present research could have applied benefits. Firstly, systematic individual measurement of the perceptual asynchronies may provide a new dimension for further characterising deficits in temporal processing in different clinical populations. For example, differences in phonological speed of processing have previously been reported in dyslexia^51^,^52^, which might result in sensory asynchronies; more generally, disruptions of temporal processing have been found in dyslexia, autism spectrum disorder and schizophrenia, characterised by differences in the temporal window of audiovisual integration^30^,^53^,^54^. As a second application, if audiovisual speech integration is reliably asynchronous in some people, then their ability to comprehend spoken words might be improved simply by artificially adjusting physical lip-to-voice asynchrony to compensate for their personal perceptual asynchrony, for example via an in-ear appliance or a software media player. To test whether any initial benefits might tend to fade due to temporal recalibration^55^, we included a further manipulation of the ordering of stimulus asynchronies, which were either held constant over short blocks, or randomised.

## Methods

### Participants

Participants were recruited via advertisement from the undergraduate population and local community. There were 36 healthy adults (26 female) aged 18 to 24 (Mean 19.7, SD 1.6). Three further participants who did not complete the second testing session were excluded from the dataset. All had normal vision and hearing by self-report. Fluent English was required, but not necessarily as a first language, given that the McGurk effect is robust across many languages^56^. Participants received either course credits or payment in return for taking part. The study protocols and use of human participants was approved by the local ethics committee at City, University of London. All participants gave informed consent, and procedures were conducted in accordance with ethical guidelines laid down in the sixth (2008) Declaration of Helsinki.

### Apparatus and stimuli

**Figure 1:**
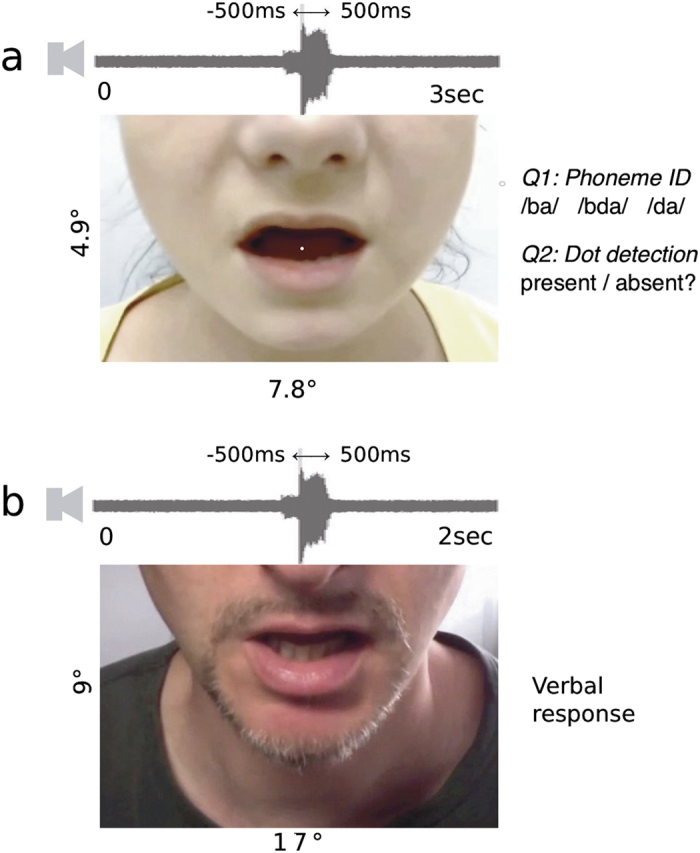
Stimuli. (**a**) McGurk task, with movie in which a white dot could occasionally appear on the tongue of the speaker, followed by two responses indicating the phoneme that was heard, and whether the dot was present or absent. Visual error feedback was provided for the second question. (**b**) Degraded Speech task, in which a movie is followed by the participant’s verbal word identification.

Visual stimuli were presented on a Sony Trinitron HMD-A420 cathode ray tube monitor. Video mode was 1280 × 1024 pixels, with 85 Hz refresh rate. Auditory stimuli were presented via two PC loudspeakers positioned on either side of the monitor. Experimental software was programmed using Psychtoolbox 3^57^ for Matlab, running on a Mac Mini. Manual responses were made via a standard PC keyboard. Viewing distance was approximately 58 cm, with head position constrained using a chin rest. Stimuli consisted of audiovisual movies depicting the lower half of a face speaking words (in the degraded speech task) or syllables (in the McGurk task). Sample images and dimensions are shown on Fig. 1. Stimuli were presented with a range of audiovisual asynchronies, spanning nine equally spaced levels from 500 ms auditory lead to 500 ms auditory lag, including simultaneous. An oscilloscope confirmed that audiovisual timing of beep and flash signals presented through the same software and hardware had minimal bias (Mean 4 ms auditory lag) and error (SD 10.30 ms).

Our choice of McGurk stimuli closely followed past studies which measured the McGurk illusion as a function of audiovisual asynchrony^31^,^35^,^58^. Phonemes /ba/ and /da/ were paired with either congruent lip movements, or with incongruent lip movements [ga] and [ba], respectively. From pilot sessions we had established that /ba/+[ga] could readily evoke the percept “da”, as the dominant McGurk fusion illusion, while for the /da/+[ba] pairing, the combination “bda” or “ba” percepts were most dominant, as reported in the above studies (and many others). For further consistency with those studies, white noise at 65 dB sound pressure level was added to the voice stimulus, with a signal-to-noise ratio of 14 dB, which might serve to enhance audiovisual interactions^1^. A small white dot (0.2 deg) could appear with 50% probability on the speaker’s tongue for 24 ms, which was used in the context of a secondary task to ensure subjects attended to the visual stimulus.

Degraded Speech stimuli consisted of two sets of 135 words selected from the MRC psycholinguistic database^59^. Search parameters included words with 4 to 6 phonemes, 5 to 8 letters, and a familiarity rating of over 400. The audio was degraded using noise vocoding through 6 logarithmically-spaced channels from 100 to 4000 Hz^60^,^61^. We determined the number of channels in prior pilot sessions to achieve a medium level of word identification difficulty. Given our interest in temporal factors associated with audiovisual speech processing, this spectral manipulation was chosen as the principle method of limiting intelligibility because it does not mask the temporal structure of the amplitude envelope^62^. However in order to create conditions more comparable to our McGurk paradigm, a constant level of low amplitude background noise was also added, in the form of a phase-scrambled version of the original voice recording with a signal-to-noise ratio of 10 dB.

### Design

There were two repeated-measures factors: task (McGurk versus Degraded Speech) and asynchrony ordering (asynchronies presented either in random order, or kept constant during a block of trials). These were combined to create four conditions, which were performed by all participants. In the Blocked conditions for both McGurk and Degraded Speech tasks, asynchronies changed incrementally every 15 trials starting with the most extreme auditory lead, and ending with the most extreme auditory lag. This was done to avoid uncontrolled fluctuations in the magnitude of aftereffects from temporal recalibration^55^ accompanying each block transition, that might otherwise occur if the sequence of blocks were randomised. It was outside the scope of this study to systematically examine any effects of auditory lag adaptation on the characteristics of the asynchrony function^63^, as these might anyway have been mixed with practice effects due to the sequential order of asynchronies. We also manipulated the retest interval as a between-subjects variable: 20 participants repeated the same task (either McGurk or Degraded Speech) twice in a single session, performing the other task a week later; the other 16 participants performed both tasks within-session, repeating them a week later. Order of task and randomisation conditions were independently counterbalanced, either within or between sessions respectively, depending on the retest-interval group.

### Procedure

Each task began with at least 15 practice trials. No performance feedback was given in either task. In the McGurk task (see Fig. 1a), each trial commenced with a fixation display. Following a key press and a blank interval (duration randomly selected from the range 1000 ms ± 500 ms), a movie was displayed for 3000 ms. The onset of the speech event was timed to start on average at 1500 ms from the start of the movie, with random jittering of ±500 ms. On each trial the stimulus pairing was selected pseudo-randomly. For the Randomised condition only, audiovisual asynchrony was also selected pseudo-randomly. A secondary task involved detecting a dot, which appeared with a probability of 50% on the speaker’s tongue at about 800 ms following movie onset (taking into account the jitter described above). Participants were instructed to attend to the mouth of the speaker in order to detect the dot, and to listen to what the speaker said.

At the end of the movie, participants were first visually prompted to indicate whether they had heard the phonemes /ba/, /bda/, or /da/, by pressing either the left, down, or right arrow keys on the keyboard. In common with previous studies of temporal functions for McGurk illusion^29^,^58^ we presented this limited number of options, which represent the percepts most typically reported for the present audiovisual combinations^31^,^64^, in order to simplify coding of percepts that were visually-driven versus auditory-driven, and thus to construct a single temporal response function. After the McGurk response, participants were then prompted to indicate whether or not they had detected a dot. Participants completed a total of 288 trials, with 8 repetitions of each of the four lip/voice combinations for each of 9 asynchronies. While the procedure of probing what was heard is common in McGurk studies, the visual probe was introduced here to encourage participants to direct their gaze and attention towards the visual lip movements, in case they become disengaged when lip-movements were highly asynchronous.

In the Degraded Speech task (Fig. 1b), a movie of a randomly selected spoken word was presented for 2000 ms. Participants then had to repeat the word verbally. The experimenter checked the response against the correct word shown on a separate display out of sight of the participant, and manually coded the response as either correct or incorrect using a separate keyboard. No feedback was provided. Participants completed 135 trials in total (15 samples of each of 9 asynchronies). A unique word stimulus was presented on each trial without repetition. For different replications of the Degraded Speech paradigm (e.g. with randomised versus blocked asynchronies), a different word set was used, with counterbalancing.

### Analysis

**Figure 2:**
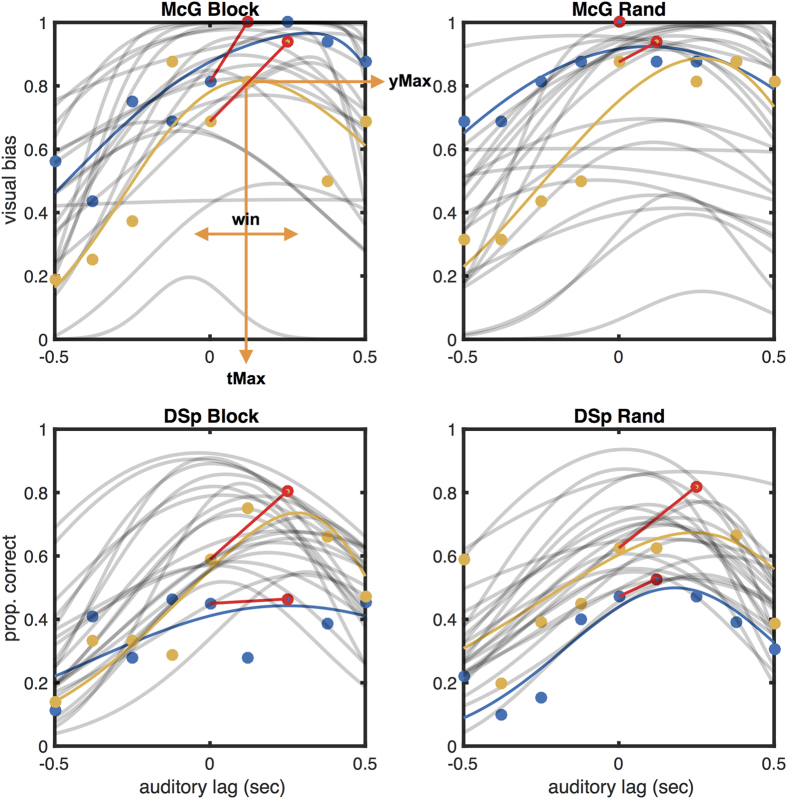
Sample raw data and best fitting functions for two participants (blue and yellow symbols), with fits for all other participants (grey lines). Separate panels for different task and conditions. Best fitting functions are shown as coloured lines for selected participant, and as light lines for all others. Orange arrows illustrate the three parameters extracted from the fitted function, and red lines and symbols illustrate the method of estimating the benefit of asynchrony for audiovisual integration from the empirical peak of the asynchrony function.

For the McGurk task we obtained the percentage of incongruent trials in which the reported phoneme was influenced by the lip movements, for each of the 9 asynchrony conditions. We coded trials as visually influenced when a ‘ba’ or ‘bda’ response was made to /da/+[ba] (11% and 24% of responses respectively, SE 0.6% and 0.2%), and a ‘da’ or ‘bda’ response was made to /ba/+[ga] (28% and 6% respectively, SE 0.1% and 0.05%). In the main analysis we combined responses to both stimulus types into an averaged measure of visual bias, but in Supplementary Materials we present results of separate analyses for each. For the Degraded Speech task, we computed the percentage of correct word identifications for each asynchrony. In both tasks these performance variables tended to rise to a peak and then fall as asynchronies varied from auditory lead to lag (Fig. 2).

We used two complementary methods to estimate the audiovisual asynchrony for maximal McGurk effect and comprehension, based either on fitting a psychometric function, or on the raw data points, the latter which required making no assumptions about the underlying temporal tuning function. For the first method (function fitting), we adopted a previously used non-monotonic asymmetric function^25^ to model task performance *y* (proportion of visually-biased responses in the McGurk effect, or accuracy of word identification in the Degraded Speech task) as it rises and falls depending on audiovisual asynchrony (*t*). Depending on its four free parameters, this function typically describes an asymmetrical bell-curve with adjustable width and asymmetry, which resembles the pattern typically formed by the empirical data when plotted against asynchrony. The function is actually composed of two Gaussian cumulative density functions (*Φ(t*), *normcdf* in Matlab, each with its own mean *M* and standard deviation *σ*). These two sub-functions, which both describe an s-shaped ascending curve, were not fitted directly to our data, but one was subtracted from the other to produce the final bell-curve (see Supplementary Figure S4 for a graphic representation), which was then compared to the data. The model equation (taken from ref. 25, their Equation 1) is therefore as follows:


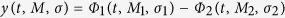


The four free parameters *M*_1_, *σ*_1_, *M*_2_, and *σ*_2_ in this equation control the width, height, asymmetry and horizontal shift of the resultant bell-curve. The goal of the function-fitting procedure was to search for the parameters that could produce a bell-curve that most closely resembled the whole pattern of observed data. We could then read off the precise location of the peak of the function along the asynchrony axis and the accuracy axis. We could also read off the values of M_1_ and M_2_ and find their relative distance, to estimate the width of the bell-curve shape. To avoid confusion, it should be understood that we did *not* fit the two separate cumulative Gaussians sub-functions (e.g. an ascending and descending function) to different sub-sets of the data directly (e.g. leftmost and rightmost halves); instead we fitted the whole asymmetrical bell-curve derived from these sub-functions to the complete distribution of data points measured across all asynchronies. This allowed us to capture individual differences in the shift of the peak relative to physical synchrony.

Fitting was performed individually for data from each participant in each condition, using an iterative maximum-likelihood Nelder-Mead simplex algorithm. The fitting procedure was programmed to run several times with a range of starting parameters, with the constraint that *M*_2_ was always greater than *M*_1_. On each iteration, the initially arbitrary shape of the CDF sub-functions would be adjusted by varying the four parameters, and then the values of one would be subtracted from the other as described by the above equation, to create a bell-curve shape. This was compared to the empirical data, and residuals were fed back to the algorithm. Iterations continued in this way until the resulting bell-curve had the optimal fit to the empirical data (i.e. the highest log likelihood ratio; the Matlab code for this was kindly shared by authors of ref. 25). Note that while this model was previously used to describe underlying theoretical constructs such as internal noise and decision criteria^25^, our own use of this model was not intended to make any particular theoretical assumptions regarding the underlying mechanisms. It was chosen mainly for the convenience of being able to obtain a variable that relates to the width of the window of audiovisual integration. This variable is conveniently obtained from the numerical distance between values of *M (M*_1_ − *M*_2_), which each relate to the position of the opposing flanks of the function on the asynchrony axis. The larger this distance, the wider and flatter the resulting bell-curve shape. Such a window width estimate is not provided by the Asymmetrical Double Sigmoid sometimes employed in previous studies^29^,^35^.

From these fitted functions, we estimated the asynchrony at which the McGurk effect or comprehension accuracy reached a peak (‘tMax’), the height of these peaks (‘yMax’), and the width of the integration window (‘Win’). Fig. 2 illustrates these parameters in relation to example fits. Data from eight participants in the McGurk condition and one in the Degraded Speech task resulted implausible parameter estimates (e.g. |tMax| > 400 ms, Win > 1500 ms. To preserve statistical power, in these cases parameter estimates were replaced by those obtained using an assumption-free method (see below), although analysis fully excluding these participants resulted in a similar pattern of correlations to those reported below. Data from three participants with estimated |tMax| greater than 400 ms (using either estimation method) were excluded from all analyses, leaving 33 participants in the final sample.

Using our second ‘assumption-free’ method we derived two dependent variables from the raw data. Firstly, for each individual we found the auditory lags required to achieve maximum visual bias in the McGurk effect, and to achieve maximum accuracy in Degraded Speech identification (tMax). For cases in which two peak data points had the same bias or accuracy, the average of their respective auditory lags was recorded as the tMax. Our second variable, ‘yMax’, simply recorded the McGurk visual bias or Degraded Speech identification accuracy at the peak data point. This method did not provide an estimate of the window width. This method of referring to the empirical peak data point was chosen for its simplicity and lack of assumptions, and also does not exclude data due to poor function fits as above, resulting potentially in greater statistical power. However, tMax estimates from the raw data lack precision as they are quantised in steps of 125 ms. To achieve greater precision, we used a bootstrapping method: for each participant and condition, the raw data from 135 trials were resampled randomly with replacement, 1000 times; tMax values were estimated from the peak datapoint of each resample and then averaged across resamples. The resulting tMax estimates varied more smoothly than the quantised original values. Pearson correlations between resampled and original estimates were high [r(30) ≥ 0.84, *p* < 0.0001]. Results and discussion of analyses using these data are reported in the Supplementary Materials.

We cross-validated these two methods by analysing the correlation of parameters estimated using fitting versus the empirical assumption-free methods described above. Correlations were highly significant [tMax: minimum r(30) = 0.78; yMax: minimum r (30) = 0.93; *p* < 0.0001].

## Results

### Dot detection

Mean dot-probe error rate in the McGurk task was 8% (Standard Error 0.6%), thus the large majority of trials were performed with attention to the lip-movements. There were no significant differences in the error rate between Random and Blocked conditions [Friedman χ^2^ = 0.011, *p* = 0.74].

### Group statistics

Group analyses were based on the empirical assumption-free data, to obtain estimates of tMax and yMax that are unbiased by model assumptions. Data from three participants were excluded as they had implausible outlying tMax values of over 400 ms of either auditory lead or lag, outside the typical range for multisensory integration^40^,^65^. On average, both tasks showed a significant bias towards auditory lags for maximising audiovisual integration: tMax for McGurk was 91 ms auditory lag (*SD* = 12.0) [t(32) = 4.38, *p* = 0.0001, two-tailed, relative to zero asynchrony], and 113 ms (*SD* = 9) for Degraded Speech [t(32) = 6.96, *p* = 0.0001]. Repeated-measures 2 × 2 ANOVA showed no significant differences in tMax or yMax between tasks, nor between Blocked and Randomised orders, and no significant interactions [maximum F(1,32) = 1.83, minimum *p = *0.19].

### Within- and between-task correlations

**Figure 3:**
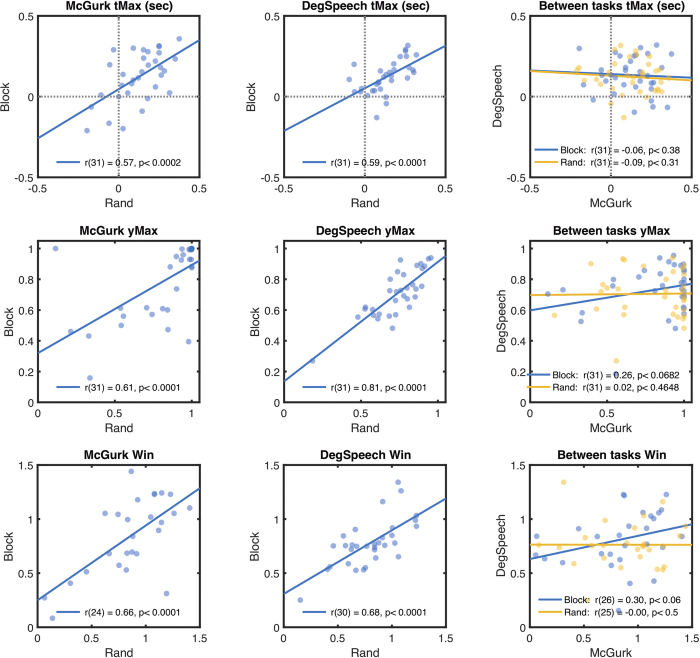
Performance measures replicate across repetitions of the same tasks, but not between different tasks. Rows of graphs respectively plot tMax, yMax and Win parameters, extracted from fitted functions. Left and middle columns respectively plot significant within-task correlations for the McGurk and Degraded Speech tasks (blocked versus random conditions). Rightmost column plots non-significant between-task correlations with separate colours for Random and Blocked conditions. Correlation statistics (one-tailed) are shown in legends.

To assess the reliability of individual differences in audiovisual timing, we measured the correlation of our dependent variables across our different conditions, estimated precisely using our fitting method. Scatterplots are shown in Fig. 3, with correlation statistics (one-tailed) displayed within each graph. Correlations between random versus blocked conditions were significant for each task [in summary, r(31) ≥ 0.57, p < 0.0005], while between-task correlations were not significant for any measure or condition [r(31) ≤ 0.26, p ≥ 0.23]. To limit the number of further analyses we used data pooled either across Degraded Speech and McGurk tasks (but keeping Random and Blocked conditions separate), or pooled across the Random and Blocked conditions (keeping the tasks separate). The within-task correlation coefficients (between Random and Blocked conditions) were significantly greater than for the between-task correlations [tMax: Fisher’s z = 2.97, *p = *0.003; yMax: z = 2.96, *p = *0.003; Win: z = 2.96, p = 0.003]. Within-task correlations were significant (one-tailed) for both same-day and next-week groups, and the respective correlation coefficients did not differ significantly depending on the retest interval [tMax same day: r(15) = 0.62, *p* = 0.004; next week: r(14) = 0.44, *p* = 0.042; Fisher’s z = 0.65, *p* = 0.52; yMax same day: r(15) = 0.76, *p* < 0.0002; next week: r(14) = 0.70, *p* = 0.0013; z = −0.76, *p* = 0.44; Win same day: r(15) = 0.46, *p < *0.03; next week: r(14) = 0.76, p < 0.0003; z(17) = −1.32, *p* = 0.19].

A very similar pattern of corresponding analyses from the assumption-free method are presented in Supplementary Materials, which may help to allay concerns that the data from the fitted data might be biased by assumptions about the shape of the underlying function. We also present a breakdown for the individual McGurk stimuli, which again showed a similar pattern of replicability.

These positive within-task correlations suggest that individual differences have persistent retest reliability, which does not depend on the retest interval. This contrasts significantly with the null correlations found between tasks, which suggest that each task depends on partially independent mechanisms, rather than generic factors such as delayed visual processing, response bias, or prior entry of auditory stimuli. In addition, these null correlations cannot be entirely attributed to measurement error given that the same data did correlate significantly within-tasks.

### Benefit of desynchronising speech

Given the above evidence that individuals differ in the asynchrony at which their speech integration is optimal, it follows that their speech integration might in principle be improved by applying an individualised auditory delay. We calculated this potential benefit by measuring how much better each individual’s speech integration is at their optimal asynchrony compared to when the stimuli are physically synchronous. We compared each individual’s empirical peak performance (yMax) with their performance as measured with synchronous stimuli (see red lines on Fig. 2). On average, the McGurk effect increased by 10.00 percentage points of visual bias (*SD* = 9.70, Median = 6.25), and Degraded Speech identification improved by 12.02 percentage points of accuracy (*SD* = 10.13, Median = 10.00). The difference in benefit between randomised and blocked conditions was neither significant for the McGurk task [Friedman χ^2^(1,32) = 1.38, *p* = 0.24], nor for the Degraded Speech task [Friedman χ^2^(1,32) = 2.13, *p* = 0.14]. There was therefore no evidence that exposure to a short train of similar asynchronies would begin to weaken any benefits of compensating for an individual’s personal asynchrony.

**Figure 4:**
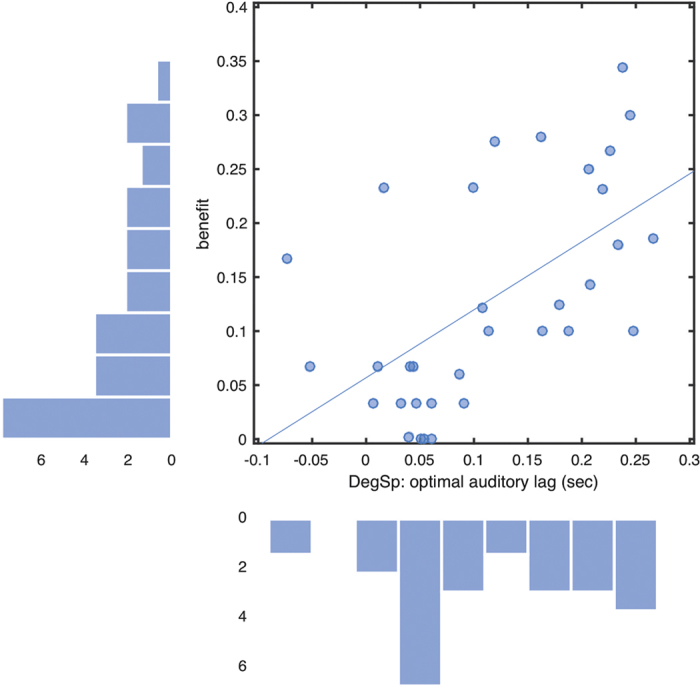
Benefit is greater for individuals showing greater audiovisual asynchrony. Benefit estimates derived from empirical (non-fitted) peak Degraded Speech (DegSp) identification accuracy (yMax), after subtracting accuracy given veridically synchronous stimuli. Vertical axis: individual benefits (units of proportion correct); horizontal axis: auditory lags for optimal identification (tMax, sec). Marginal histograms show the distribution of benefits (vertical) and optimal asynchronies (horizontal).

Note that our method of extracting data from the peak across asynchronies (see Analysis section above) makes no implicit assumptions about the underlying function. We therefore wished to test whether assumption-free estimates of tMax and yMax measures both actually relate to an underlying asynchrony tuning function that is shifted towards different asynchronies in different individuals and tasks. We reasoned that the more such a function is shifted away from simultaneity, the better performance would be at the peak of the function compared to when measured at zero auditory lag, because performance would be sampled from successively lower flanks of the implicit tuning function relative to the peak. This predicts that our above measure of ‘benefit’ should correlate significantly with increasing values of tMax. This prediction was confirmed [Block: r(31) = 0.39, one-tailed *p* = 0.012; Rand: r(31) = 0.57, *p* = 0.0003; averaged across conditions: r(31) = 0.58, *p* = 0.0002, see Fig. 4]. Thus, the more an individual’s vision subjectively lags their audition, the greater the benefit is likely to be from correcting for this by artificially delaying the audio (Fig. 4). Note that if all underlying functions were actually centred on synchrony, and variations in tMax occurred merely due to the presence of random peaks, then no such correlation should have been found.

## Discussion

This research has returned to an age-old debate over whether vision and audition are out of synch. While past explicit subjective measurements of sensory asynchronies might have been prone to generic higher-level attentional influences or decisional biases, we have used implicit and bias-resistant methods to measure audiovisual speech integration as a function of audiovisual asynchrony. Our results support the view that perceptual asynchronies are real rather than an artefact of bias, and reflect individually stable but task-specific processing delays. These delays can be estimated using our methodology to define a ‘Personal Equation’ quantifying each individual’s asynchrony. Our results further suggest that speech integration is suboptimal for naturally synchronous stimuli in many of the individuals that we tested, and that artificial correction of each individual’s perceptual asynchrony might improve their multisensory integration. This may lead to concrete therapeutic applications, for example improving speech comprehension by delaying voice relative to lip movements.

McGurk and Degraded Speech tasks have sometimes been used interchangeably^46^, in line with studies which have found evidence for common mechanisms driving correlations between alternative measures of the window of subjective synchrony^66^ and speech integration^46^. It is therefore interesting that here, tMax and yMax measures only correlated reliably across repetitions of the same task, not between McGurk and Degraded Speech tasks. This suggests that we may have not just one general ‘Personal Equation’, but different ones for different tasks. This null correlation is unlikely to be an artefact of measurement error because the identical data were evidently reliable enough to correlate significantly across replications of the same task; moreover, these within-task and between-task measures of association were significantly different to one other. The between-task null correlation is also arguably not attributable exclusively to stimulus differences in the temporal structure of congruent versus incongruent audiovisual pairings, for these would be more likely to affect all participants in a similar way, rather than each participant in a different way. There were other differences in the stimuli, for example the degraded speech task involved a different multisyllabic word for each trial, while the McGurk task repeated the same small set of phonemes. However, if the pattern of performance were so sensitive to the precise phonemic structure the verbal stimuli, then the data for the Degraded Speech might be much noisier, with poor replicability, because the data are composed of trials that each relate to unique stimuli, compared to the McGurk task, where the identical stimuli were repeated over many trials. The visual angle also differed between stimuli, however the most informative mouth regions were still positioned centrally in the display, and any systematic differences in visual processing time for peripheral stimuli would likely have affected mean tMax rather than resulting in uncorrelated individual differences. Thus, the null correlation between McGurk and Degraded Speech measurements seems unlikely to reflect noisy measurement due to differences in stimulus characteristics between the tasks.

Instead of supporting common mechanisms or stimulus differences, our results seem more consistent with past evidence for functional dissociations in performance depending on the task, observed for example in detection versus identification^45^, or the stimuli used, for example meaningful words versus meaningless syllables^46^,^67^. More specifically, fMRI evidence suggests that congruent and incongruent audiovisual speech involve distinct brain areas, in Superior Temporal Sulcus and posterior Superior Temporal Gyrus, respectively^47^,^49^. These areas were proposed to support distinct functions of first binding and then merging the incongruent auditory and visual stimuli^48^, respectively. In particular it has been proposed that detection and comprehension of audiovisual speech in noise relies on a domain-general audiovisual binding mechanism which exploits temporal covariation of crossmodal signals^3^,^68^, whereas the integration of phonetic codes generated by auditory and visual speech recruits a distinct speech-specific mechanism^45^, which may provide a substrate for the McGurk illusion. The present results imply that such mechanisms may be not only functionally and anatomically distinct, but also temporally independent in terms of the dynamics of their response to incoming stimuli.

This evidence of independence follows previous reports of uncorrelated measures of audiovisual timing based on explicit judgements of temporal order versus synchrony^18^,^19^,^20^,^21^, which have been inferred to probe independent mechanisms. However decisional factors might have contributed to variance between explicit measures^18^,^20^,^22^,^23^, which implicit measures are more immune to. In addition, the observation of independence between these implicit measures is inconsistent with ‘prior entry’ as a sufficient explanation of perceptual asynchronies, whereby processing of information in one modality can be accelerated or delayed relative to the other depending on the allocation of attention^15^,^16^,^24^. Such a general attentional bias towards vision versus audition might be expected to affect all audiovisual tasks similarly, resulting in a positive correlation of PEs across tasks, but that pattern was not evident here. It might still be argued that the predicted positive correlation might have been masked by differing attentional biases for McGurk and Degraded Speech tasks. However our implicit measures of integration should be less susceptible to attentional biases because they depend on processes that are thought to be largely pre-attentive and immune to manipulations of attention to specific modalities^36^,^37^,^38^, at least under the conditions tested here^69^,^70^. Furthermore, we found no evidence of consistent shifts in average tMax between tasks that might be expected if each made differing attentional demands. In summary, our new evidence of independent timing for different tasks does not support a general attentional ‘prior-entry’ bias as a sufficient explanation of non-zero tMax estimates and their individual variability.

Our results agree with several past studies, which have reported asymmetrical or shifted average asynchrony functions with greater tolerance for auditory lags than for auditory leads or even a preference for auditory lags, using congruent or incongruent (McGurk) speech or other naturalistic stimuli, and explicit or implicit measures^30^,^41^,^58^,^66^,^71^,^72^,^73^. Our results go further by revealing the variability and stability of individual preferences for auditory lags. Some of the variability might be explained given that incongruent McGurk-type stimuli may make it difficult to determine when lips and voice are actually perceived as synchronous relative to veridical synchrony, however we found comparable (though uncorrelated) variability using precisely timed congruent stimuli. The speed of light relative to sound might play a minor role in accounting for the bias towards auditory lags^8^, although the physical delay between modalities is relatively small at typical social distances relevant to speech (e.g. ~3 ms at 1 m) compared to some of the tMax values we observed. An alternative explanation of the average preference for auditory lags could be that lip movements might be used predictively to disambiguate phonemes because they typically precede audible phonemes (e.g. lips puckering before uttering plosive consonants^3^,^29^,^45^,^46^,^74^,^75^, which might even speed up auditory processing^72^,^76^). If so, average tMax values might relate less directly to the point at which auditory and visual information are perceptually synchronous than to the temporal statistics of the stimuli. However, while the availability of such predictive visual information might explain tolerance or even preference for auditory lags in both tasks on average, the wide but replicable individual variability of tMax values, and their null correlation between tasks, seems inconsistent with a common influence from the predictive temporal structure of the speech stimuli, which would have been the same for all participants.

These considerations lead us to conclude that individual differences in tMax and their correlational patterns may reflect temporal variables intrinsic to different parts of the perceptual system. For example, there may be variable latencies for propagation and processing neural signals as they converge on the different multimodal sites responsible for different tasks^10^,^17^,^27^,^50^,^77^ via connections that may vary in length and efficiency across sites and individuals. Whilst the relative arrival time of auditory and visual signals at their respective unisensory cortices is only on the order of 28 ms^10^,^77^, it is possible that additional latencies are accrued as signals propagate towards multimodal areas and are subsequently processed within them, resulting in the average critical auditory delays measured here (100 ms, SD = 69 ms, Median = 875 ms).

An attractive neurophysiological framework for explaining our findings might be provided by the idea that auditory signals are maximally amplified by visual signals when they converge during the high-excitability phase of ongoing neural oscillations in auditory cortex^78^. Indeed, perceived relative timing of auditory versus visual events may even relate quite directly to the phase of such neural signals relative to the ongoing oscillatory cycle^79^. Within this framework, the observed individual differences in the optimal asynchrony for integration might be explained in terms of local misalignments of the phase of signals within different brain networks representing the visual and auditory inputs, both relative to each other and to the broader pattern of oscillations emerging across the whole ensemble.

Our earlier work^35^ provides further support for such a view in which neural and perceptual timing are assumed to be closely related^17^,^44^,^50^,^80^. We previously compared tMax for McGurk with the Point of Subjective Simultaneity derived from temporal order judgements (‘*did lips move before or after you heard the voice?*’), both under dual-task^35^ and single-task contexts using the same stimuli^81^. Surprisingly, these measures correlated *negatively*. Thus the more one task was subject to, say, a visual lag, the more the other task appeared subject to an auditory lag. This counterintuitive effect led us to propose a theory of Temporal Renormalisation^35^, whereby the neural timing within one brain sub-network (e.g. responsible for integrating audiovisual speech) is normalised *relative to* the neural timing of corresponding events across an ensemble of other independent sub-networks (which would include those specifically responsible for subjective temporal judgements). However, on the neural oscillations account above, renormalisation might only manifest when the ensemble and the individual sub-networks are all reacting to stimuli that have a similar temporal structure, so that the phases of oscillations remain consistent across stimulation episodes. This could explain why we only found a negative correlation previously where different tasks were based on the same stimuli, but not in the present study where very different stimuli were used for the different tasks.

An applied implication of our findings is that speech comprehension (and possibly other abilities) might be optimised by delaying the auditory stimulus (or video for some individuals) by the precise amount needed to raise an individual’s performance to the peak of their asynchrony tuning function, thus effectively resynchronising their audiovisual integration. This might be achieved via a modified hearing aid or software media player with a programmable auditory or visual delay. For half of our sample, stimulation at an individual’s optimal asynchrony resulted in an average improvement of word identification by 20 words in every 100. Encouragingly, we found no evidence that such benefit decays when the asynchrony is prolonged for short blocks of trials. Benefits among hearing-impaired individuals might be even greater, due to their greater reliance on visual speech cues^4^. However these measurements were made with simple syllables and words, under variable asynchronies. Future research will need to measure any benefits of consistent asynchronies for understanding more complex sentences and natural conversation.

More generally, our findings imply that sensory asynchronies can influence higher cognitive abilities such as speech comprehension, resulting in sub-optimal integration of naturally synchronous stimuli even in young neuro-typical subjects. It is therefore important to measure across a range of asynchronies, when assessing the ability to integrate information across modalities. Past studies have observed large variability in susceptibility to McGurk effect^82^, but this might be due to differences in sensory timing rather than efficiency of integration; indeed many of our participants showed very high McGurk susceptibility when the stimuli were presented at their individually optimal asynchrony. Whether such sensory asynchronies may contribute to differences in cognitive performance, across the normal lifespan, and in conditions such as dyslexia^51^,^52^,^81^ and schizophrenia^30^, are interesting questions now amenable for study using unbiased measurements of sensory asynchronies of the kind presented here.

In conclusion, our study has shed new light on a controversy that has continued for over two centuries^11^,^12^,^13^ over the interpretation of apparent individual differences in sensory asynchrony. While traditional subjective measures are often prone to generic attentional or decisional biases, our new bias-resistant objective measures now allow us to discount such biases as a sufficient explanation. Consequently, our measures may be informative about real asynchronies present in perceptual processing. Such asynchronies could cause a real problem: given that lip-voice integration and speech comprehension were often at their best with a slight auditory lag in some of our neurotypical participants, their perception might actually be suboptimal with naturally synchronous vision and audition. Fortunately, now that we can robustly measure perceptual asynchrony and find each individual’s ‘Personal Equation’, we may be able to artificially correct for it and thus potentially improve speech comprehension.

## Additional Information

**How to cite this article:** Ipser, A. *et al*. Sight and sound persistently out of synch: stable individual differences in audiovisual synchronisation revealed by implicit measures of lip-voice integration. *Sci. Rep.* **7**, 46413; doi: 10.1038/srep46413 (2017).

**Publisher's note:** Springer Nature remains neutral with regard to jurisdictional claims in published maps and institutional affiliations.

## Supplementary Material

Supplementary Materials
