# Supplementary material for: Sight and sound persistently out of synch: stable individual differences in audiovisual synchronisation revealed by implicit measures of lip-voice integration
Source: Sci Rep. 2017 Apr 21;7:46413. doi: 10.1038/srep46413 (PMC5399466; doi:10.1038/srep46413)
Supplement: Supplementary Materials [file srep46413-s1.doc]

**Supplementary Information**

**Sight and sound persistently out of synch:
stable individual differences in audiovisual synchronisation revealed by implicit measures of lip-voice integration.**

**Alberta Ipser**

**Vlera Agolli**

**Anisa Bajraktari**

**Fatimah Al-Alawi**

**Nurfitriani Djaafara**

**Elliot D Freeman**

# Within and between-task correlations (assumption-free analysis)

To address concerns that the pattern of results from the fitted data might be biased by assumptions about the shape of the underlying function, we used an alternative ‘assumption-free’ method to estimate tMax and yMax based directly on the empirical data. We simply read off the auditory lags required to achieve maximum visual bias in the McGurk effect, and to achieve maximum accuracy in Degraded Speech identification. For cases in which two peak data points had the same bias or accuracy, the average of their respective auditory lags was recorded as the tMax. Five participants in the McGurk task had gaps between peaks larger than 500ms; median gap was 188ms; there were no such cases in the Degraded Speech task (median 0ms). Nevertheless, the replicability of McGurk results across sessions (see below) suggests that the presence of multiple peaks in the raw data may represent a relatively stable characteristic of perceptual timing, rather than just a statistical artefact.

We then used a bootstrapping procedure to resample the original empirical asynchrony tuning functions to generate 1000 new profiles (see Figure S1); each of these new profiles could have different peaks due to sampling error, and thus a slightly different tMax. Averaging across these new tMax values could help to reduce bias from this sampling error, and also provides greater numerical precision than the original values which varied only in steps of 125ms. One concern using bootstrapping is that the sample is truly representative of the population that is to be estimated. True representativeness is hard to establish here as we have only these restricted samples. However, datapoints on each asynchrony function do tend to form a consistent inverted-U shape as should be expected from the population; this underlying function is further validated by our observation that 'benefit' increases with the 'tMax' estimated using the bootstrapping method, which would not be found if tMax were based on purely random variations (see main results). Furthermore, the successful cross-validation against tMax derived from the fitted procedure (see main results) improves our confidence that the bootstrapping method can extract intrinsic properties of the data rather than amplifying statistical artefacts.

We performed within-task and between-task correlations using the parameter estimates derived from the assumption-free method based on the empirical peak of the temporal tuning function (see Method reported in main text; note that the window of integration parameter could not be estimated using this method). The pattern of results was very similar to the analyses based on function-fitting method reported in the main text (compare Figure S2 with Figure 3 in main text). As mentioned in the main text, tMax estimates correlated very highly between the two measures.

There were significant positive correlations (one-tailed hypotheses throughout) between Random and Blocked asynchrony orderings when compared within each task, both for tMax [McGurk: r(31) = 0.40, *p* < 0.01; Degraded Speech: r(31) = 0.68, *p* < 0.00005; data averaged across tasks: r(31) = 0.57, *p* < 0.0005], and for yMax [McGurk: r(31) = 0.52, *p* = 0.0009; Degraded Speech: r(31) = 0.72, *p* < 0.00005; data averaged across tasks: r(31) = 0.63, *p* < 0.00005]. Particularly for the McGurk effect, the illusion was often nearly 100% likely at participants’ optimal asynchrony, resulting in many high yMax values. However the within-task correlation was still significant after excluding participants with such ceiling yMax [r(12) = 0.58, *p* = 0.015].

Figure S1

Sample datasets from four participants in the Degraded Speech task (separate rows) showing raw identification accuracy (red circles and lines), bootstrapped data (blue lines) and bootstrapped peaks (small red dots). Red crosshairs indicate the average peak based on bootstrapping, with 95% confidence intervals for tMax shown as horizontal errorbars.


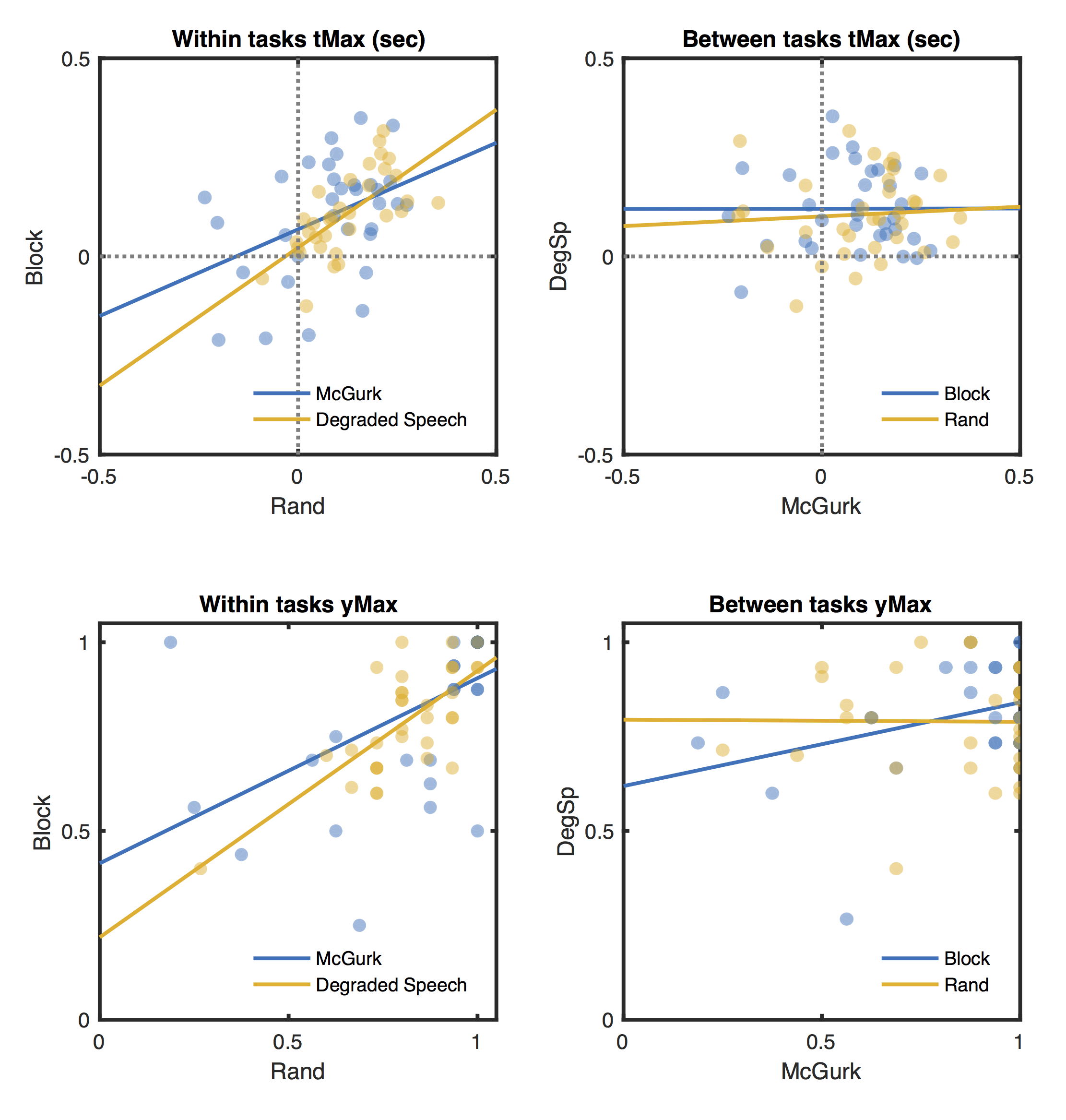


Figure S2

Scatterplots showing correlations within tasks (left graphs with separate colours for McGurk and Degraded Speech tasks) and between tasks (rightmost column with separate colours for Random and Blocked conditions). Separate rows of graphs are shown for each of tMax and yMax parameters.

# Breakdown of responses for the McGurk task

We calculated proportions of three different response types for each audiovisual combination in the McGurk task, and for each block-duration and randomisation condition, averaged across asynchrony. Results are shown in Figure S3 as stacked bars. As expected, the ‘bda’ response was made most frequently in the /da/ + visual [ba] condition, on average 22% of the time (SE 1.7%). Conversely, in the auditory /ba/ + visual [ga] combination, ‘da’ was the most dominant, as expected (mean 26%, SE <1%), while 'bda' was the least frequent response on average (mean 7%, SE 1%) of the time. This latter response might reflect an ambiguous percept between /ba/ and /da/ categories. As it deviated from pure /ba/, we coded this as visually-driven. However, as the frequency was fairly low, this is unlikely to have had much impact on the results.

As in the analysis reported in the main text, we examined the replicability of tMax and yMax values across repetitions of the McGurk task but broken down by each audiovisual combination (using fitted data). For /ba/+[ga], tMax correlated significantly across repetitions [r(32) = 0.55, *p* = 0.0004, one-tailed throughout], and so did yMax [r(32) = 0.65, *p* < 0.0001]. For /da/+[ba] the correlation was borderline significant for tMax [r(32) = 0.27, *p* < 0.06], and significant for yMax [r(32) = 0.70, *p* < 0.0001]. tMax showed a null correlation between two different audiovisual combinations [Block: r(30) = 0.20, *p* = 0.15; Random: r(30) = 0.10, *p* = 0.30], while yMax values did correlate significantly [Block: r(30) = 0.65, *p*< 0.0001; Random: r(30) = 0.37, p = 0.002]. This is broadly consistent with previous suggestions that processing of ‘fusion’ and ‘combination’ illusions evoked by McGurk stimuli may be subject to different temporal constraints (Martin et al, 2013).

Figure S3

Stacked bar chart showing the proportion of each response type, ‘ba’, ‘da’, and ‘bda’, for each audiovisual combination of /phonemes/ with [lip movements] in the McGurk task.

# Fitting procedure

Figure S4

Illustration of the asymmetrical bell-curve function *y*(t) (red), which we used to model how performance changed with audiovisual asynchrony t. This function was derived from the subtraction of two cumulative Gaussian sub-functions (dotted lines), each with their own two free parameters 𝜎 and M (vertical lines). These parameters were adjusted iteratively until the resulting bell-curve provided a maximum-likelihood fit to the empirical data (red circles, from one example participant). Note that the individual CDFs are not fitted directly, but are combined via subtraction to create the bell-curve which is then compared to the observed data.
